# The Relationship Between Business Environment and Single Champion Enterprise Entrepreneurship

**DOI:** 10.3389/fpsyg.2021.788053

**Published:** 2022-01-11

**Authors:** Chanti Wu, Jinjin Lin

**Affiliations:** School of Business Administration, Ningbo University of Finance and Economics, Ningbo, China

**Keywords:** business environment, institutional configuration, fuzzy set qualitative comparative analysis (fsQCA), manufacturing single champion, single champion enterprise entrepreneurship

## Abstract

What kind of business environment can produce high single champion enterprise entrepreneurship is a new issue for discussion in research on entrepreneurship. Based on institutional configuration theory and the fcQCA method, the present paper analyses the relationship between the business environment and single champion enterprise entrepreneurship from the perspective of configuration. This paper studies the role of the business environment in 80 case cities all over the country in promoting high single champion enterprise entrepreneurship and discusses three business environment configurations concerning high single champion enterprise entrepreneurship and two configurations concerning non-high single champion enterprise entrepreneurship. Three typical business environment element configurations can promote high single champion enterprise entrepreneurship, namely, the market innovation type dominated by multiple resources, the financial service–driven type assisted by resources, and the market-driven type led by financial services, which reflects the significance of financial services and the market environment.

## Introduction

In 2016, the Ministry of Industry and Information Technology issued the *Implementation Scheme for Special Action for Cultivation and Promotion of Manufacturing Single Champion Enterprises*, which guided manufacturing enterprises to focus on innovation and quality improvement and play a leading role as champion enterprises in the global market and technology in more segmented markets. At present, the global manufacturing pattern is being revolutionized. The United States, Japan, and other developed economies continue to attract the reshoring of high-end manufacturing, and the Southeast Asia region is scrambling for international industry transfer by virtue of its labor force advantage. Due to the coronavirus disease 2019 (COVID-19) epidemic, global economies have suffered an exogenous shock never seen before with a strong economic and psychosocial impact on organizations (De et al., 2021). In the face of new changes in international industry development, a group of “single manufacturing champion enterprises,” which focus on “intensive cultivation” in segmented industries and fields and reach the height of global or national champion, are becoming the leading power occupying industrial development leadership, commanding heights, and promoting high-quality development of manufacturing representing the highest development level and the strongest market strength in the global or national segmented industry field. However, how to draw lessons from growth and cultivation of single champion and stably and continuously cultivate manufacturing single champion enterprises in the face of the new development pattern of “domestic major cycle as the main body and domestic and international cycle as the mutual promotion?” What kind of challenges are faced by single champion enterprises in China led by international strategies at the time of achieving great success? How do single champion enterprises start businesses and make progress with the supply side reform–environmental change of dual cycle pattern system? How do entrepreneurial antecedent variables, such as market, product, technology, of single champion enterprises collide and coexist with the new institutional environment, and how do single champion enterprises adapt to the entrepreneurial environment of the dual cycle pattern dominated by the domestic cycle, which need to be resolved immediately?

Research on single champion enterprises are still in their infancy without a corresponding theoretical pattern that can totally interpret entrepreneurial growth and the environment of single champion enterprises in China so far. Under the impact of global COVID-19, single champion enterprises have sprung up and bucked the trend and should be taken as typical enterprise research objects as shown in Table 1. In combination with institutional theory, qualitative and quantitative methods are comprehensively leveraged for theoretical review and analysis, and the influencing mechanism of institutional reform on entrepreneurship evolution of single champion enterprises is meticulously revealed. At the government level, continuous cultivation and healthy growth of single champion enterprises have practical significance in promoting high-quality development of economy in China, enhancing the advanced level of manufacturing in China and advancing construction pilot and demonstration cities of the “Made in China 2025 Strategy.” At the enterprise level, the specific entrepreneurial environment of single champion enterprises and new institutional environment ecology can help enterprises propose and utilize internal elements, control external elements, improve management efficiency, and promote high-quality development of enterprises.

**TABLE 1:** Top 10 provinces in the number of manufacturing single champion demonstration enterprises.

Province	The first batch	The second bath	The third batch	The fourth batch	The fifth batch	Total	Ranking
Shandong	12	12	16	20	24	84	1
Zhejiang	6	14	11	15	21	67	2
Jiangsu	7	8	14	10	10	49	3
Guangdong	6	3	3	7	6	25	4
Fujian	2	6	4	2	3	17	5
Beijing	1	3	1	3	5	13	6
Henan	3	4	4	1	1	13	6
Anhui	3	2	4	–	2	11	8
Liaoning	2	3	1	–	4	10	9
Hubei	2	2	4	–	1	9	10

## Literature Overview

### Research on Single Champion

Manufacturing single champion enterprises refer to those enterprises that focus on some specific segmented product markets in the manufacturing industry for a long time, possess international leading production technology or processes and single product market occupation leading in the world. At present, relatively less literature is related to single champion enterprise research in China (Chengda, 2019), so this paper focused on research on high-tech and hidden enterprises. Entrepreneurial growth research on enterprises is the focus of domestic and foreign economics and management research, and different scholars have attempted to specify the growth impetus elements of enterprises with theory and empirical research. It is generally believed that the impetus can be divided into internal and external impetuses, and impetus elements of enterprise growth mainly included resources (Chengda, 2019), knowledge (Mingjie, 2006), capacity (Zhun and Guoshun, 2008), technology (Bottazzi, 2001), and government policy (Thorsten, 2002). Domestic and foreign scholars focus on explanation and analysis from analyzing factors affecting high-tech enterprise growth and pay attention to the enterprise growth concept, influencing factors, influencing modes, and paths and enterprises boundaries. Actual institutional environment issues and policies of single champion enterprises are short of research.

### Institutional Configuration Theory

The institutional theory holds that results are produced by multiple factors. Multiple institutional configurations can result in high entrepreneurial activeness of enterprises with functionally equivalent paths (Furnari et al., 2020). Diversified institutional logic form in the business environment ecology exerted an effect on the environment carrying capacity and entrepreneurship efficiency of service-driven enterprises (Aldrich and Ruef, 2006). In 2001, after the “business environment” was formally proposed, domestic and foreign scholars carried out qualitative and quantitative research. Optimization of the business environment to promote entrepreneurial activeness came into the focus of entrepreneurship research (Yunzhou et al., 2020).

### Research on the Business Environment

The business environment refers to the integrated ecosystem of the external environment for entrepreneurship and other activities by enterprises. Government efficiency, human resources, financial environment, public services, market environment, and innovation environment should be integrated into account in the business environment ecology (Zhijun et al., 2019).

Government efficiency is essential for the external growth impetus of single champion enterprises. Government efficiency not only represents the administrative service level of local government (Guo and Tian, 2021), but also the transaction cost of local government (Yunzhou et al., 2020). It can significantly affect economic behaviors in some regions (Whitley, 1999). High government efficiency can help lower enterprise operation costs and improve operation efficiency so as to promote enterprise growth.

Human resources determine the supply of human capital of single champion enterprises. As the core resources of enterprises, human resources are conducive to improving productivity and promoting innovative growth of enterprises by direct participation in production and operation (Sun et al., 2021). The level of human resources is helpful in increasing the possibility of enterprises in regions to obtain core resources and promote the growth of enterprises (Ye Wenping et al., 2018).

Financial service level affects the financing cost of single champion enterprises. Higher review of financial services in regions would help with enterprise operation (Mei and Song, 2021). A good financial system and financing cost can contribute to advancing the growth of single champion cultivation enterprises (Xin et al., 2019).

The level of public services embodies the level of infrastructure services for meeting people’s lives, survival, and development and other direct demands. It exerts an important influence on resources of production and operation activities of enterprises in regions as well as selection of investment areas (Zhijun et al., 2019). Research shows that infrastructure can lower the production cost of the manufacturing industry (Guangnan and Ran, 2013) and advance the growth of single champion enterprises.

As an intangible tool, the market can allocate important resources. The regional market environment can directly determine consumption of products produced by enterprises and market demand scale (Zhijun et al., 2019). Research shows that a highly open market environment can reduce market entry barriers, create a sound market environment, and promote enterprise growth.

The innovation environment mainly reflects innovation input, innovation cooperation, technology spillover, and learning effects to promote the innovation performance of enterprises (Zhijun et al., 2019). Innovation is a new combination of production factors (Yunzhou, 2019; Du and Kim, 2021). A high innovation environment can facilitate enterprise competition, expand the innovative ecological environment, and promote benign growth of enterprises. The rise of digital and interconnected technology within the workplace, including programs that facilitate monitoring and surveillance of employees, is unstoppable (Kalischko and Riedl, 2021).

In conclusion, it can be known from research conclusions of scholars on entrepreneurial and business environments of single champion enterprises that the majority of research analyses and discusses from the point of enterprise development modes and evolution paths and focuses on the influence and function of technology, innovation, industry cluster, and other development on enterprises. However, present research on the entrepreneurial environment of single champion enterprises should be brought to attention due to the lack of systematic and deep theoretical and empirical research. Although much research is being carried out from the angle of entrepreneurship theory of enterprises at present, empirical research in specific regions is not combined, not to mention little research as regards to institutional theory. It is necessary to carry out theoretical and empirical specific research on entrepreneurship development of single champion enterprises to explore changes in their entrepreneurial environment.

## Research Design

### Research Method

The present paper adopts fuzzy set qualitative comparative analysis (fsQCA) to explore the causal complex mechanism of the urban business environment on entrepreneurial growth of urban single champion enterprises. fsQCA is based on Boolean algebra and set theory to combine qualitative and quantitative analysis as a whole (Ragin, 2008) to find the causal relationship between condition configuration and results through comparison between cases (Douglas et al., 2020), which is applicable for multiple concurrent causal problems (Yunzhou and Liangding, 2017; Jianqing et al., 2019; Feifei and Fei, 2020). The fsQCA method goes especially well with research on the ecological element combination of business environment and causal mechanism of entrepreneurial growth of urban single champion enterprises in this paper.

Compared with traditional qualitative and quantitative analysis, the fsQCA method is advantageous in the following aspects: The traditional regression analysis method is usually used to handle symmetric correlation but cannot be used to explain an asymmetric causal relationship in the real world. The fsQCA method is more commonly used for complex real relationships and logics because it does not assume symmetry of the causal relationship in advance. The fsQCA method breaks through the limitation of logic assumed in traditional statistical methods to take a holistic view of the complex impact of multiple causes on outcomes. It is believed that different combinations of elements generate the same results without the requirements on control variables. fsQCA requires high flexibility in sample size. This paper studies 80 case cities, which comprise a medium sample size. Sample size restricts the effective development of qualitative and quantitative analysis but meets the requirements of the fsQCA method on sample quantity.

### Data Sources

Research data in this paper mainly come from the following two aspects. First, data on the antecedent variable, urban business environment, come from *Evaluation of Urban Business Environment in China* (Zhijun et al., 2019), in which six business environment index elements of four municipalities, five municipalities with independent planning status, 27 provincial capitals, and 254 prefecture-level cities were analyzed, among which six elements were government efficiency, human resources, financial services, public services, market environment, and innovation environment, respectively. The main data in the report came from the “China City Database” and “China Urban-Rural Construction Database” in the EPS global statistics data/analysis platform, and statistical data of each city from 2015 to 2016 were used (Zhijun et al., 2019). Second, data on the outcome variable, entrepreneurial growth of urban single champion enterprises, came from *List of the First Batch of Manufacturing Single Champion Demonstration (Cultivation) Enterprises* and *List of the Second Batch of Manufacturing Single Champion Demonstration (Cultivation) Enterprises* issued by the Ministry of Industry and Information Technology in 2017 and *List of the Third Batch of Single Champion Enterprises and Manufacturing Single Champion Products* issued by the Ministry of Industry and Information Technology in 2018 to screen case cities with single champion demonstration cities in manufacturing as shown in Table 2. Finally, after Bazhou City, which lacked urban business environment data, was eliminated following data matching, 80 cities with complete data were taken as research cases. In addition, the research was combined with authoritative media reports in cities and governments’ official websites to further qualitatively analyze the configuration found through fcQCA.

**TABLE 2:** Regional distribution information on cases.

Regional distribution	Number of cities	Number of enterprises	Regional distribution	Number of cities	Number of enterprises
Shandong	12	40	Hunan	2	3
Zhejiang	7	31	Shaanxi	2	3
Jiangsu	10	28	Shanxi	2	2
Fujian	6	12	Tianjin	1	2
Henan	6	11	Jiangxi	2	2
Guangdong	4	12	Chongqing	1	1
Anhui	7	9	Xinjiang	1	1
Hubei	3	8	Yunnan	1	1
Beijing	1	6	Guizhou	1	1
Liaoning	2	5	Heilongjiang	1	1
Sichuan	2	4	Jilin	1	1
Hebei	4	3	Inner Mongolia	1	1
Shanghai	1	3			
**Total**				**80**	**191**

### Variable Measurement

#### Result Variable

Entrepreneurship of urban single champion enterprises refers to the quantity of single champion demonstration start-ups accumulated in the city, namely, the quantity of single champion demonstration enterprises in manufacturing in the city. Enterprise entrepreneurship between (1, 12) was produced. The closer the value was to 12, the better the entrepreneurship of single champion enterprises in the city.

#### Antecedent Condition

In this paper, measurement was carried out in accordance with six business environment elements in *Evaluation of Urban Business Environment in China*. Every business environment element index includes 17 level II indexes as shown in Table 3 in detail. Level II index data are expressed as data on provinces, districts, and cities directly obtained. The utility value method was adopted when making indexes dimensionless, and the range of utility value was (0, 100), which means the optimal utility value under the index was 100, and the worst utility value was 0 (Zhijun, 2019).

**TABLE 3:** Index evaluation system of antecedent condition.

Level-I indexes	Level-II indexes	Weight (%)	Basic data source
Government efficiency (0.15)	Average budgetary expenditure (RMB 10,000)	50	China City Database
	Government service efficiency	50	Chinese Local Government Efficiency Research Report
Human resources (0.2)	Average salary level (RMB)	40	China City Database
	College enrollment (person)	30	China City Database
	Number of organization jobholders at year end (10,000)	30	China City Database
Financial services (0.15)	Private financing efficiency (RMB 10,000)	50	China City Database
	Overall financing efficiency (RMB 10,000)	50	
Public service (0.2)	Road area per capita (square meter per person)	15	China Urban-Rural Construction Database
	Water supply capacity (10,000 tons)	25	China Urban-Rural Construction Database
	Gas supply capacity (10,000 m^3^)	25	China Urban-Rural Construction Database
	Power supply capacity (10,000 KWH)	25	China Urban-Rural Construction Database
	Medical and health service (sheet per 10,000)	10	China City Database
Market environment (0.2)	Per-capita GDP (RMB)	40	China City Database
	Gross investment in fixed assets (RMB 10,000)	30	China City Database
	Amount of foreign capital actually used that year (RMB 10,000)	30	China City Database
Innovation environment (0.1)	Scientific expenditure (RMB 10,000)	50	China City Database
	Innovation capacity index	50	Report on Innovation in Chinese cities and Industries

#### Variable Calibration

Variable calibration refers to fuzzy variables that converts conventional variables between zero and one. This paper adopted fsQCA 3.0 software for data calibration and three calibration points totally affiliated to, crossing with, and totally unaffiliated to six condition variables and one result variable was, respectively, set as the upper (75%), mid, and lower quartile (25%) for descriptive statistics of the case sample. Non-high enterprise entrepreneurship growth was calibrated by taking the non-set of high entrepreneurial growth. The calibration anchor and descriptive statistics of each variable are shown in Table 4 in detail.

**TABLE 4:** Set, calibration and descriptive statistics.

	Calibration of fuzzy set			Descriptive analysis			
Set	Totally unaffiliated	Crossing with	Totally affiliated	Average value	Standard deviation	The minimum value	The maximum value
Entrepreneurship	1.000	2.000	3.000	2.388	1.978	1.000	12.000
Government efficiency	5.723	7.900	13.860	12.613	13.787	0.830	80.400
Human resources	11.945	16.830	28.750	23.121	16.238	6.560	80.180
Financial services	2.083	4.590	14.270	11.531	17.010	0.540	100.000
Public services	9.203	13.160	18.670	16.894	12.830	3.740	67.480
Market environment	11.473	18.360	27.870	21.386	13.102	3.810	72.420
Innovation environment	0.965	2.510	5.710	7.153	15.145	0.150	85.410

## Research Results

### Analysis of Necessary Conditions

Before the truth table procedure analysis of the fuzzy set, it must be checked whether the antecedent variable is necessary for the result variable, generally measured with consistency and coverage rate. Consistency is manifested as the degree to which the result variable requires the antecedent variable, whereas the coverage rate manifests as interpretable sample size that could interpret the necessity of the antecedent variable. In general, in case consistency is more than 0.9, the antecedent condition is considered necessary and conforming to inspection standards of necessary conditions. Table 5 shows consistency and coverage rate results of antecedent condition necessity for the result variable, among which consistency is smaller than 0.9 and is not required.

**TABLE 5:** Necessity test of single condition.

Condition variable	High enterprise entrepreneurship		Condition variable	Non-high enterprise entrepreneurship	
	Consistency	Coverage rate		Consistency	Coverage rate
Government efficiency	0.744	0.659	Non-government efficiency	0.699	0.778
Human resources	0.748	0.661	Non-human resources	0.701	0.781
Financial services	0.777	0.699	Non-financial services	0.739	0.810
Public services	0.769	0.689	Non-public services	0.729	0.802
Market environment	0.799	0.700	Non-market environment	0.733	0.824
Innovation environment	0.786	0.710	Non-innovation environment	0.750	0.818

This paper adopts fsQCA 3.0 software to, respectively, analyze the environment configuration that results in high single champion enterprise entrepreneurship and non-high single champion enterprise entrepreneurship. These different configurations represent business environments in which two different results are realized (Yunzhou et al., 2020). Configurations found in this paper are named based on configuration theory process (Furnari et al., 2020). In this paper, six antecedent variables all met consistency check standards, and two result variables were not subject to important necessary conditions. Therefore, no conditions were eliminated in this paper, and all variables were placed in the true table for analysis.

### Institutional Environment Producing High Single Champion Enterprise Entrepreneurship

In this paper, the original consistency threshold was set as 0.75, the PRI consistency threshold as 0.70, and the case frequency threshold as 1. Through standard analysis, complex, intermediate, and simple solutions were generated. In the complex solution, no logic residue was used with multiple constituent forms of results and relatively complex path analysis. As for the simple solution, all logic residues were used. Due to unassessed rationality and too simplified necessary conditions, result formation was not realistic. As for the intermediate solution, meaningful logic residues were included into the solution according to research theory and practical knowledge, and necessary conditions were not allowed to be eliminated. Data result analysis is shown in Table 6, in which ZF, RL, JR, GG, SC, and CX, respectively, represent government efficiency, human resources, financial services, public services, market environment, innovation environment, and other antecedent variables.

**TABLE 6:** Data results.

High enterprise entrepreneurship	Solutions	Configuration	Original coverage rate	Net coverage rate	Consistency	Overall coverage rate	Overall consistency
	Complex solution	∼ZF*RL*GG*SC*CX RL*JR*GG*SC*CX ∼ZF*∼RL*JR*∼GG*SC*∼CX	0.190 0.605 0.092	0.007 0.423 0.035	0.835 0.795 0.920	0.647	0.791
	Intermediate solution	∼ZF*RL*GG*SC*CX RL*JR*GG*SC*CX ∼ZF*∼RL*JR*∼GG*SC*∼CX	0.190 0.605 0.092	0.007 0.423 0.035	0.835 0.795 0.920	0.647	0.791
	Simple solution	∼ZF*JR RL*GG*SC*CX	0.250 0.619	0.068 0.437	0.766 0.789	0.687	0.762
Non-high enterprise entrepreneurship	Complex solution	∼RL*∼JR*∼GG*∼SC*∼CX ∼ZF*∼JR*∼GG*∼SC*∼CX	0.545 0.520	0.052 0.027	0.882 0.905	0.572	0.886
	Intermediate solution	∼RL*∼JR*∼GG*∼SC*∼CX ∼ZF*∼JR*∼GG*∼SC*∼CX	0.545 0.520	0.052 0.027	0.882 0.905	0.572	0.886
	Simple solution	∼JR*∼GG*∼SC*∼CX	0.586	0.586	0.887	0.586	0.887

“∼” expresses “non”. “*” expresses “multiplication”.

Table 7 shows subject analysis results for realizing high and non-high single champion enterprise entrepreneurship. It can be known from Table 7 that three institutional configurations, namely, G1, G2, and G3, exist for the realization of high single champion enterprise entrepreneurship with an overall coverage rate of 79.1% and overall consistency of 64.7%, which shows that the configurations can be used to explain 64.7% of high single champion enterprise entrepreneurship and 79.1% cases. The configuration and realization of high single champion enterprise entrepreneurship constitute a perfect subset that can highly interpret the realization of high single champion enterprise entrepreneurship.

**TABLE 7:** Configuration to realize high and non-high single champion enterprise entrepreneurship.

	High enterprise entrepreneurship			Non-high enterprise entrepreneurship	
Conditions	G1	G2	G3	FG1	FG2
Government efficiency	∘		○		∘
Human resources		•	∘	∘	
Financial services	•	∙	•	○	○
Public services	•	•	∘	○	○
Market environment	•	•	∙	○	○
Innovation environment	•	•	∘	○	○
Consistency	0.835	0.795	0.920	0.886	0.905
Original coverage rate	0.190	0.605	0.092	0.545	0.520
Unique coverage rate	0.007	0.423	0.035	0.052	0.027
Overall consistency	0.647			0.572	
Overall coverage rate	0.791			0.886	

*Core and non-core elements of configuration are distinguished in combination with simple solutions and intermediate solutions in this paper. Here, • indicates the existence of core conditions, and ○ indicates the shortage of core conditions; ∙ indicates the existence of marginal conditions, and ∘ indicates the shortage of marginal conditions. Blank indicates core conditions or marginal conditions might or might not occur.*

#### Market Innovation Type Dominated by Multiple Resources

It is pointed out in configuration G1 that high financial services, high public services, high market environment, and high innovation environment are core conditions, which supplements the marketing environment in which the non-high government condition is taken as a marginal condition and can generate high single champion enterprise entrepreneurship. This paper also finds that entrepreneurship of single champion enterprises is different from that of small and medium-sized enterprises, and high financial environment and public service level can effectively promote single champion enterprise entrepreneurship in cities without high government, and high market, and innovation environments. Typical cities with such a business environment include Zhenjiang and Yantai. According to business environment data statistics, due to smaller scales, government efficiencies in Zhenjiang and Yantai are inferior to those of municipalities with independent planning status or other large cities. These two cities have a human resource level below the middle but perform well in financial services, public services, market environment, and innovation environment. Since 2016, as a “Benefiting-enterprise Pioneer,” the Yantai Bureau of Industry and Information Technology has positively driven enterprise development and promoted transformation, upgrading, and accelerated leaping development of enterprises. Zhenjiang City has served its entity economy with finance and promoted its vigorous improvement and development through connecting with financial capital of Shanghai.

#### Financial Service–Driven Type Assisted by Resources

It is pointed out in configuration G2 that high human resources, high public services, high market environment, and high innovation environment are core conditions, which supplement the marketing environment in which high finance is taken as a marginal condition and can generate high single champion enterprise entrepreneurship. This paper finds that, led by the market environment, human resources, public environment, and other well-resourced high innovation environments can promote single champion enterprise entrepreneurship. Typical cities with such business environments include Ningbo, Qingdao, and Wuxi. According to business environment data statistics, Ningbo, Qingdao, and Wuxi have good market environments, higher overall innovation environments, and sufficient urban public services and human resources. Financial services in these three cities are gradually developing. Taking Ningbo as an example, as of January 2021, a total of 22 single champion demonstration enterprises have occurred, ranking in first place all over China. In the recent 3 years, three single champion cultivation enterprises have developed into demonstration enterprises in Ningbo City, ranking in first place all over the country.

#### Market-Driven Type Led by Financial Services

It is pointed out in configuration G3 that high financial services and non-high government efficiency are core conditions that supplement the marketing environment in which non-high human resources, non-high public services, non-high innovation environment, and high market environment are taken as marginal conditions and generate high single champion enterprise entrepreneurship. In cities with non-high government environment and high financial service environment, high market environment support can effectively support single champion enterprise entrepreneurship. Taizhou is a typical city with such a business environment. According to business environment data statistics, Taizhou City focuses on serving industry transformation, real economy, and financial industry development. At the same time, it fully promotes the construction of a national financial reform test area, makes full use of the “vital role” of finance to maintain fundamentals of market entity, and explores the use of capital to accelerate industrial transformation and development.

### Institutional Environment Generating Urban Non-high Single Champion Enterprise Entrepreneurship

It can be known from Table 7 that two institutional configurations, namely, FG1 and FG2, can realize urban non-high single champion enterprise entrepreneurship with an overall coverage rate of 88.6% and overall consistency of 57.2%, which shows that configuration can explain 57.2% of realization of non-high single champion enterprise entrepreneurship and 88.6% cases. Configuration and realization of non-high single champion enterprise entrepreneurship constitute a perfect subset that can highly interpret the realization of non-high single champion enterprise entrepreneurship. Configuration FG1 and FG2 show that, under non-high public services, non-high financial services, non-high market environment and non-high innovation environment, high single champion enterprise entrepreneurship is unlikely to occur. Different from middle and small-sized enterprise entrepreneurship, single champion enterprise innovation requires high-quality financial services, public service supports, and an institutional element configuration with a stable and healthy market environment and active innovation environment, which is less dependent on government and human resources.

### Robustness Test

In this paper, robustness tests were carried out for antecedent configuration of high single champion enterprise entrepreneurship (Ming and Yunzhou, 2019). First, the threshold of case number was increased to two, resulting in a configuration that was basically consistent. Second, PRI consistency was increased from 0.70 to 0.75, resulting in a configuration that was basically consistent. Finally, in consideration of differences in institutional element resource level between cities, after separately deleting municipalities, municipalities with independent planning status, and first-tier cities, analysis results were basically consistent. Robustness test showed that results were robust.

## Conclusion and Prospects

### Research Conclusion

The influence of the business environment on single champion enterprise entrepreneurship is the new focus of entrepreneurship research. Based on an institutional configuration framework, this paper discusses the relationship between the business environment and single champion enterprise entrepreneurship from the perspective of configuration with fcQCA method. This paper studies the role of the business environment in 80 case cities all over the country in promoting high single champion enterprise entrepreneurship and discusses three business environment configurations of high single champion enterprise entrepreneurship and two configurations of non-high single champion enterprise entrepreneurship (Feng et al., 2021; Li et al., 2021). Three typical business environment element configurations can promote high single champion enterprise entrepreneurship, namely, the market innovation type led by multiple resources, the financial service–driven type assisted by resources, and the market-driven type led by financial services, which reflect the significance of financial services and the market environment (Bian et al., 2021).

### Shortage and Prospects

Due to a shortage of research data sources and limited time, data sources in 2018 are only used for the antecedent and result variables, and quantity of single champion demonstration enterprises in cities before 2018 is utilized for the result variable. In future research, data on five groups of single champion demonstration enterprises can be combined to study the dynamic evolution mechanism of the business environment on single champion demonstration enterprise entrepreneurship. In addition, this paper discusses the influence of the business environment in cities all over the country on single champion demonstration enterprise entrepreneurship. In following research, typical cases in which enterprises are cultivated into demonstration enterprises can be used for configuration analysis to explore what kind of business environment can promote the high-quality and high-efficiency entrepreneurship of single champion enterprises.

## Data Availability Statement

The raw data supporting the conclusions of this article will be made available by the authors, without undue reservation.

## Ethics Statement

The studies involving human participants were reviewed and approved by the Ningbo University of Finance and Economics Ethics Committee. The patients/participants provided their written informed consent to participate in this study. Written informed consent was obtained from the individual(s) for the publication of any potentially identifiable images or data included in this article.

## Author Contributions

Both authors listed have made a substantial, direct, and intellectual contribution to the work, and approved it for publication.

## Conflict of Interest

The authors declare that the research was conducted in the absence of any commercial or financial relationships that could be construed as a potential conflict of interest.

## Publisher’s Note

All claims expressed in this article are solely those of the authors and do not necessarily represent those of their affiliated organizations, or those of the publisher, the editors and the reviewers. Any product that may be evaluated in this article, or claim that may be made by its manufacturer, is not guaranteed or endorsed by the publisher.
